# Novel Orthoreovirus from Diseased Crow, Finland

**DOI:** 10.3201/eid1312.070394

**Published:** 2007-12

**Authors:** Eili Huhtamo, Nathalie Y. Uzcátegui, Tytti Manni, Riggert Munsterhjelm, Markus Brummer-Korvenkontio, Antti Vaheri, Olli Vapalahti

**Affiliations:** * Haartman Institute–University of Helsinki, Helsinki, Finland; † Tvärminne Zoological Station–University of Helsinki, Hanko, Finland; ‡ HUSLAB Hospital District of Helsinki and Uusimaa, Helsinki, Finland; § Faculty of Veterinary Medicine–University of Helsinki, Helsinki, Finland

**Keywords:** Orthoreovirus, crow, isolate, avian disease, West Nile virus, letter

**To the Editor:** Corvids, especially American crows (*Corvus brachyrhynchos*), are reported to be highly susceptible to lineage 1 of West Nile virus (WNV), which causes them to show symptoms of encephalitis. They are regarded as indicator species in the surveillance of WNV in the United States (*1*). In parts of Europe, WNV is endemic and studies are ongoing to detect WNV in wild birds. Thus far, no evidence of WNV in birds has been found in northern Europe.

In August 2002, in southern Finland, a diseased wild hooded crow (*Corvus corone cornix*) was found flying abnormally with coordination problems, abnormal postures, cramps, and paralysis. Because WNV infection was suspected, virologic tests were performed, which resulted in the isolation of a novel orthoreovirus, which was likely the causative agent of the disease.

Avian orthoreoviruses (ARVs) belong to the family *Reoviridae*, genus *Orthoreovirus*. They infect wild and farm-raised birds and are important fowl pathogens associated with various disease conditions such as gastrointestinal malabsorption syndrome, tenosynovitis (arthritis), growth retardation, and sudden death. They have also been isolated from asymptomatic birds. The reovirus virion is icosahedral, nonenveloped, and has a double-capsid structure that shelters the segmented double-stranded RNA genome (*2*).

Heart, lung, liver, kidney, and brain tissues of the diseased crow tested negative for WNV RNA. Virus isolation from brain homogenate was carried out in BHK (baby hamster kidney)–21 cells. On day 2 after infection, a strong cytopathic effect was observed, including syncytium formation. Spherical, spiked virus particles, consistent with those of members of the family *Reoviridae,* were observed by electron microscopy. The diameter of the particles was slightly smaller (≈70 nm) than that reported for ARV (85 nm) (*3*). Members of the genus *Orthoreovirus* differ in their host reservoir and capability of syncytium formation; most avian orthoreoviruses are fusogenic and fail to agglutinate erythrocytes, unlike the mammalian reoviruses (*4*). The isolate, designated as Tvärminne avian virus (TVAV), failed to hemagglutinate chicken, goose, or human O erythrocytes.

Members of the genus *Orthoreovirus* have a genome consisting of 10 dsRNA segments in 3 size classes, large (L1–3), medium (M1–3), and small (S1–4). The RNA was extracted from TVAV-infected BHK-21 cells with TriPure isolation reagent (Roche Diagnostics, GmbH, Mannheim, Germany). Ten double-stranded RNA genome segments were separated by electrophoresis, showing a pattern typical of ARV with the S1 segment migrating between S- and M-segment classes (*5*). The S1 segment encodes the orthoreovirus type-specific antigen, σC protein, which is the minor outer-capsid protein, a spiked structure mediating cell attachment.

For phylogenetic analyses, the partial σC gene was amplified by reverse transcription–PCR with avian reovirus–specific primers (*6*). The obtained sequence (GenBank accession no. DQ470139) was aligned with 25 published orthoreovirus sequences. The phylogenetic tree was constructed by using the maximum likelihood method, with general-time reversible model of substitution determined by Modeltest using PAUP* (*7*). The analyses showed that TVAV did not group with avian or mammalian orthoreoviruses but formed a separate clade (Figure). In further analysis, no evidence for recombination events was found. The nucleotide sequence homology of the σC gene was <50%, and amino acid homology was <40%, when compared with previously described orthoreovirus strains. Additionally, a partial M3 segment was sequenced (GenBank accession no. EU053426) that also showed low (<40%) amino acid homology and genetic relation to other orthoreoviruses, which supports the result obtained from the σC gene.

**Figure:**
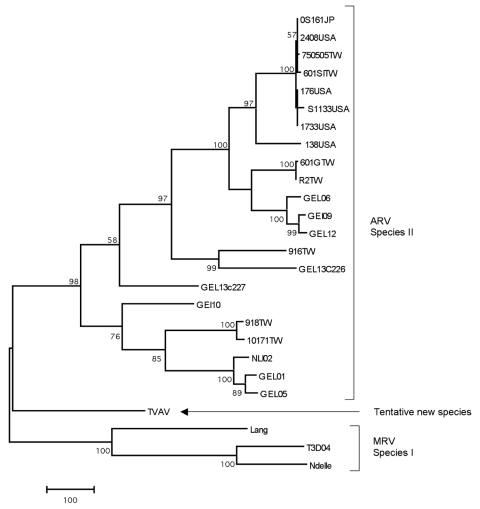
Maximum parsimony tree based on a 916-bp nucleotide sequence of the σC gene. The scale bar indicates a branch length corresponding to 100 character-state changes. Bootstrap support values <50 are not shown. The tentative species is shown together with the closest relatives within the *Orthoreovirus* genus; avian orthoreovirus (ARV), mammalian orthoreovirus (MRV). GenBank accession nos.: AF204946, AF204945, AF204950, AF204947, AF18358, L39002, AF004857, AF218359, AF297217, AF297213, AF354224, AF354220, AF354225, AF297214, AF354226, AF354227, AF354219, AF297215, AF297216, AF354229, AF354221, AF354223, DQ470139, M10260, AY785910, AF368035**.**

To our knowledge, no sequences of ARV isolates have been previously available from northern Europe. The TVAV isolate described differs clearly from other known ARV strains and could be considered a candidate for a new species in the genus *Orthoreovirus*. ARVs are not generally associated with encephalitic disease, in contrast to reoviruses that infect mice, baboons, and snakes (*8*,*9*). Systemic infection with ARV could cause viremia also in the brain, but since other tissues were not studied, whether they were infected remains unclear. In Finland, a bird-pathogenic orthoreovirus was isolated in the same geographic region 6 years earlier from the bursa of Fabricius from common eider (*Somateria mollissima*) carcasses and was suspected to be the cause of their death (*10*). The eider reovirus induced syncytium formation, lacked hemagglutination activity, and had an RNA genome segment migration pattern similar to that of TVAV. However, instead of showing symptoms that appeared to affect the central nervous system, experimentally infected mallards (*Anas platyrhynchos*) showed hemorrhages in liver, spleen, and bursa of Fabricius tissues. Unfortunately, no sequence data are available from the eider virus isolate that can be compared with TVAV. Because many ARVs are poultry pathogens of economic importance, more studies are needed to determine the taxonomic classification of the TVAV isolate and its pathogenicity for avian hosts. In addition, the recognition of potential avian pathogens in wild birds is important due to the possible threat for farm-raised birds and also for the surveillance of zoonotic viruses transmissible to humans.
